# Crosstalk-Reduced Double-Layer Half-Divided Volume Holographic Concentrator for Solar Energy Concentration

**DOI:** 10.3390/s20236903

**Published:** 2020-12-03

**Authors:** Hongxu Kao, Jianshe Ma, Chengchen Wang, Taihui Wu, Ping Su

**Affiliations:** Tsinghua Shenzhen International Graduate School, Tsinghua University, Shenzhen 518055, China; khx17@tsinghua.org.cn (H.K.); ma.jianshe@sz.tsinghua.edu.cn (J.M.); chengche19@mails.tsinghua.edu.cn (C.W.); wuth18@mails.tsinghua.edu.cn (T.W.)

**Keywords:** solar energy, volume holographic concentrator, volume-phase holograms, large-angle solar concentrating, photopolymer

## Abstract

A new double-layer sunlight concentration system, where each layer is divided into two regions, is proposed, and the system has four volume holograms. Since the four holograms convert light in different directions, the interlayer crosstalk is reduced, and the system has a high concentration ratio. The simulation results show that the concentration system can achieve a 30° operation angle range. The holograms are fabricated on photopolymer substrates, and the left half of the system is implemented using two holograms. The characteristics of the left half of the system are assessed. The agreement of the simulation and experimental results on diffraction efficiency validates the proposed method. The tested monochromatic concentration ratio can achieve a record of 418.8, and the concentration ratio under sunlight is 5.38. The experiment results of light use efficiency are close to the simulation with non-crosstalk, which indicates that the interlayer crosstalk is small.

## 1. Introduction

Solar energy is a kind of clean energy that can be recycled. The existing solar energy utilization technology is generally realized by laying photovoltaic (PV) cells [1,2,3] on a large area. However, its cost is higher than other energy generation, such as hydropower, gas, coal, fuel oil and nuclear power. In order to solve this problem, solar concentrating technology is used to reduce the area of PV cells, so as to reduce the cost and improve the efficiency of solar energy utilization. Solar concentrating elements can be classified into traditional solar concentrators and holographic solar concentrators [4,5]. A traditional photovoltaic solar concentrator includes a focus element and a tracking system, wherein the focus element is usually a Fresnel lens. A Fresnel lens usually has a very small working angle (<1°), so a tracking system to accurately track the position of the sun is essential [6,7]. Since the optical axis needs to be parallel to the incident direction of sunlight, the structure is relatively complicated, and the manufacturing and maintenance costs are relatively high. In addition, the system can only work normally in direct sunlight, and the effect of solar focusing is poor in cloudy weather.

In contrast, a holographic solar concentrator usually has a much larger working angle. It can not only receive the normal incident solar light, but it can also receive the oblique incident light at a certain angle, which improves the utilization efficiency of solar energy, and does not need a tracking system. A holographic solar concentrator has the advantages of low cost, high efficiency, a large receiving angle and a simple system structure. A holographic solar concentrator can reduce the area of expensive PV cells by depositing inexpensive thin film over large areas [8]. Holographic solar concentrators are normally thin and lightweight optical components used in variety of applications, e.g., in optical sensing systems [9], optical fiber sensing systems [10] and multitudinous holographic concentrators [11]. One kind of holographic solar concentrator, the volume-phase hologram (VPH), can collect sunlight from a large area and focus the light onto a smaller area, which can effectively reduce the area of the sunlight receiving devices. Therefore, the cost of the solar energy concentration systems can be greatly reduced.

For large-angle solar energy collection, VPH large-angle solar concentration devices have received much attention in recent years, due to high diffraction efficiency and low cost of VPH [6,12]. The most commonly used materials for VPH solar concentration elements are dichromatic gelatin (DCG) and photopolymer [13], which have high diffraction efficiency, permissible multilayer integration and adjustable dispersion. However, a single layer of VPH can only receive a small incident angle range of sunlight. To make high-efficiency, large-angle, wide-band response and low-dispersion concentration systems, VPHs are often cascaded, combined with reflection, divided into different function areas or recorded with angle multiplexing.

To design holographic solar concentrators, one factor that needs to consider is the position of the sun. Ludamn first proposed an implementation for large-angle solar concentration using VPHs in 1982 [14]. Since then, the reflection and transmission VPHs are widely used in solar concentration systems. Iurevych et al. designed a system to improve solar energy conversion efficiency [15], and this system uses internal reflection and diffraction by a VPH to realize photoelectric and photothermal conversion. Segundo et al. presented a reflection VPH solar energy concentration system [16], in which the sunlight was concentrated on the PV cells at large angles by VPH diffraction and total internal reflection. Subsequently, a recording method for VPH by using angle multiplex and iterative interference was proposed by Lee et al. [17]. Experimental results showed that the system can concentrate incident light from three different angle ranges on the same position with low diffraction efficiency.

A multi-layer concentration system formed by cascading VPH can obtain a larger operation angle range without tracking. However, in a multi-layer concentration system, if the diffraction angle of the top layer is within the operation angle range of the lower layer, unexpected diffraction by the lower layer happens, and the efficiency of the system decreases. To reduce this interlayer crosstalk, Castro et al. [18] proposed a double-layer VPH solar concentration system, where the two layers diffracted light into opposite directions. Although crosstalk is reduced, the concentration ratio of the system is only about 1.5, because the system only uses linear holographic gratings.

Martin et al. designed another multi-layer structure using VPH for solar energy concentration to reduce inter-layer crosstalk [19]. The system achieves 18° total concentration angle range with a monochromatic concentration ratio of 20. However, there is another kind of crosstalk due to the linear holographic gratings. Subsequently, a single-layer VPH concentration system was designed by Bianco et al. [20]. The concentration ratio of sunlight was 5.85, and the operation angle range of the system was 60°. However, only one region could work normally when the sunlight incident within the operation angle of the system, so the diffraction efficiency was one third of the diffraction efficiency of one single layer without division. Yanru Jiang et al. [21] designed a new multi wavelength Fresnel lens to focus light, in order to improve the utilization rate of solar energy. This method improved the uniform irradiance of solar cells and the conversion efficiency. However, the disadvantage of this design is that the receiving angle of Fresnel concentrator is less than ±1°, which cannot achieve large angle solar concentrating.

To achieve a larger focus angle range, higher concentration ratio and diffraction efficiency, we propose a new design method of VPH concentration system, based on Kogelnik’s coupled wave theory [22]. The design method can optimize the number of holographic gratings. According to this method, we propose a double layer, half-divided sunlight concentration system, which is proven to have high diffraction efficiency and concentration ratio. To reduce interlayer crosstalk, we employ only convergent VPHs. Each layer is halved to achieve trade-offs between operation angle range and diffraction efficiency. The volume holographic large-angle solar concentrating technology used in this paper does not need to accurately track the position of the sun, which reduces the system cost and further reduces the solar energy utilization cost. Due to its large acceptance angle (>10°), it can still work in rainy weather with scattered light, which is beneficial to the promotion and use in areas with fewer sunny days within a year. Compared with other large-angle light-concentrating technologies, the volume holographic film in the volume holographic high-angle light-concentrating technology has the advantages of easy replication, low price and strong replaceability.

This paper consists of five sections. In Section 2, the design method is presented, and the new sunlight concentration system is designed and modeled. In Section 3, the recording procedure and conditions are described. In Section 4, the optical properties of the system are tested and compared to the simulation results. Section 5 provides the conclusion.

## 2. Design Method

Normally, the whole visible spectrum of the sun needs to be collected. The relative location between the sun and a certain point on the earth varies during a day. Therefore, the VPH for a sunlight concentrator must have a certain operation angle and wavelength range, while having a reasonable diffraction efficiency. When designing a sunlight concentration system, it is important to consider the trade-offs between an operation angle or wavelength range and diffraction efficiency. Inspired by the previous work [14,15,16,17,18,19,20], we describe the new design method in this section. First, some basics of VPH are stated, and then the modeling and design method are proposed.

### 2.1. Basics about Volume Phase Holograms

**Figure 1:**
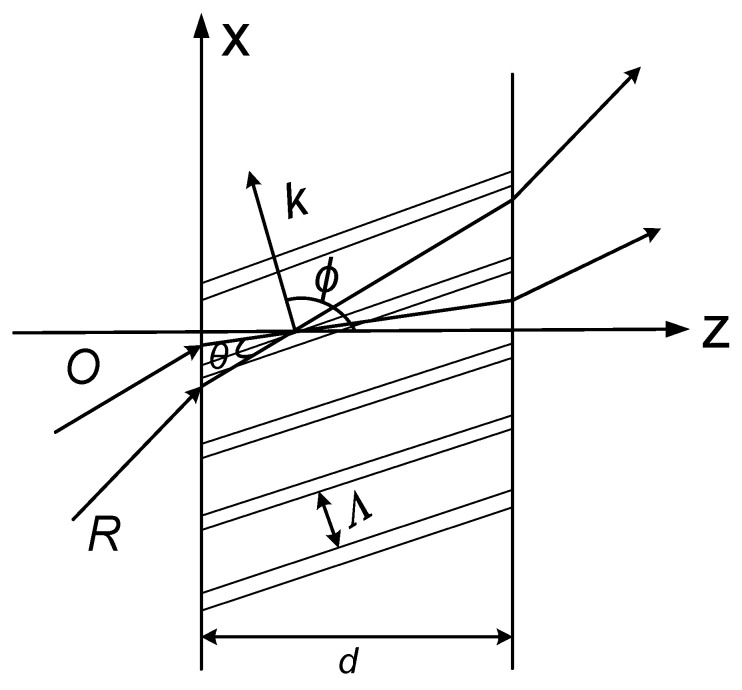
Schematic diagrams of recording the transmission hologram. O: object wave; R: reference wave; θ is the angle between the object light and the reference light, referred to as the Bragg angle, Λ is the volume holographic grating constant; ϕ is the tilt angle of the grating fringe; k=2π/Λ is the wave number.

As shown in Figure 1, an object wave and a reference wave interfere in a medium of thickness d to modulate periodically the refractive index of the medium and form a hologram. If the Q value is larger than 10, then the recorded hologram is a VPH [23,24]

(1) $$Q=\frac{2\pi \lambda_{s}}{n\Lambda^{2}},$$

where $\lambda_{s}$ is the wavelength of the incident light in the medium with the refractive index n, which is related to the wavelength $\lambda_{0}$ of the light in the vacuum by $\lambda_{s}=\lambda_{0}/n$. d is the thickness of the holographic material substrate, Λ is the grating constant, which is the reciprocal of spatial frequency.

If a wave is irradiated onto a VPH and the following Bragg condition in Equation (2) is satisfied, the diffracted wave has the strongest energy:

(2) 2Λsinθ=λ,

where θ is the angle between the object light and the reference light, referred to as the Bragg angle, Λ is the volume holographic grating constant and λ is the wavelength of the incident light wave.

When incident light deviates from the Bragg angle, the diffraction efficiency drops. Similarly, any incident wavelength deviating from the Bragg condition also causes a drop of diffraction efficiency. Those two phenomena are called angular and wavelength selectivity of VPH. To calculate the diffraction efficiency under different incident wavelengths and angles, we employ an approximate coupled-wave algorithm.

**Figure 2:**
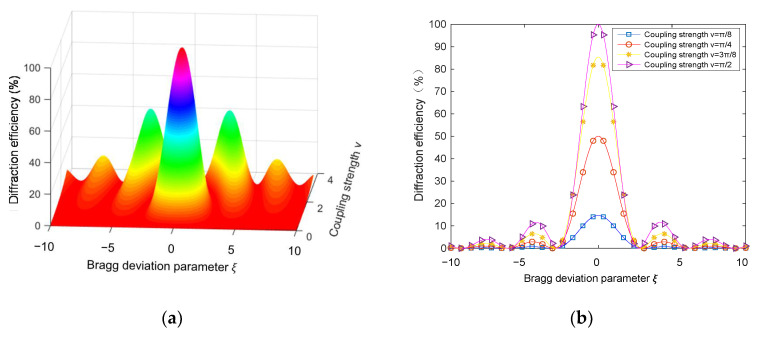
Variation of non−absorptive transmission volume hologram diffraction efficiency $\eta_{T}$ with: (**a**) coupling strength ν and Bragg deviation parameter ξ, (**b**) Bragg deviation parameter ξ under coupling strength $\nu =\frac{\pi }{8},\frac{\pi }{4},\frac{3\pi }{8},\frac{\pi }{2}$.

The most widely used approximate coupled-wave algorithm for VPH is the Kogelnik coupled wave theory [22]. In Kogelnik coupled wave theory, the effect of VPH is considered to be the energy exchange process between the incident wave and the diffracted wave. The diffraction efficiency is defined as the ratio of the diffracted wave’s intensity to the incident wave’s intensity, where the absorption by the holographic material is ignored. According to Kogelnik coupled wave theory, the diffraction efficiency of a transmission volume phase hologram can be expressed by

(3) $$\eta_{T}=\frac{sin^{2}{\left(\nu^{2}+\xi^{2}\right)}^{1/2}}{1+\xi^{2}/\nu^{2}}$$

where ξ is the Bragg deviation parameter of the incident wave, $\xi =\Delta \theta \mathrm{kd}\sin\left(\phi -\theta \right)/2c_{S}-\Delta \lambda k^{2}d/8\pi nc_{S}$. The parameter ν=πΔnd/λcosθ is the volume holographic coupling strength, where Δn is the refractive index modulation, and it is an important parameter for forming a VPH. The parameter $c_{s}$ is the tilt factor of the object light and ϕ is the tilt angle of the grating fringe, as shown in Figure 1. The parameter Δθ is the angular offset and Δλ is the wavelength offset, and these two parameters reflect the angular selectivity and wavelength selectivity, respectively. The parameter k=2π/Λ is the wave number. The relationship between the non-absorptive transmission volume hologram diffraction efficiency $\eta_{T}$ with the Bragg deviation parameter ξ and the volume hologram coupling strength ν is shown in Figure 2a. If the Bragg condition (ξ=0) is fully satisfied, diffraction efficiency reaches the maximum. However, as the absolute value of the Bragg deviation parameter increases, the diffraction efficiency decreases rapidly. When |ξ| reaches a certain value, the diffraction efficiency is zero. When |ξ| is increased, the diffraction efficiency can still exhibit small fluctuations. However, the peak values of the diffraction efficiency curve can decrease gradually. Figure 2a clearly shows the angle and wavelength selectivity property of a VPH.

Figure 2b shows the curves of hologram diffraction efficiency with varying Bragg deviation parameter ξ when *ν* equals *π*/8, *π*/4, 3*π*/8 and *π*/2. As seen from Figure 2b, the coupling strength ν affects not only the gradient of the diffraction efficiency but also the peaks of the diffraction efficiency. Therefore, after the selection of holographic material, the operation angle and wavelength range should be optimized by studying the angular and wavelength selectivity by using Kogelnik coupled wave theory.

### 2.2. Studying of Wavelength and Angular Selectivity

**Figure 3:**
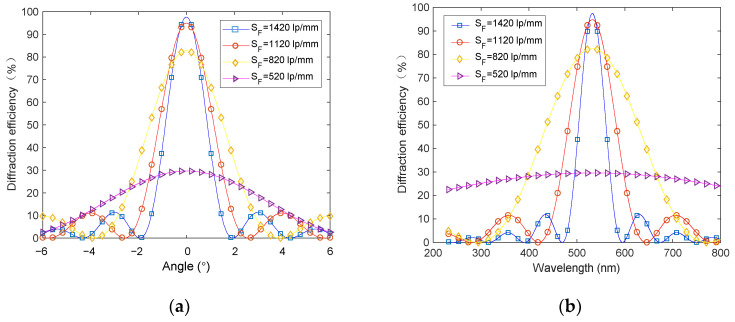
Selectivity of volume hologram under different spatial frequencies: (**a**) angle selectivity and (**b**) wavelength selectivity.

The photopolymer Bayfol HX200 [25,26,27,28] was used as the volume holographic grating’s recording material. The sensitive wavelength of this material covers the range of 400 to 700 nm, which is suitable for solar light concentration, the thickness is 16 μm, and the average refractive index is 1.5. To record a volume hologram, by substituting Q≥10, d = 16 μm, n = 1.5 into Equation (1), we can calculate the spatial frequency $S_{F}$ ≥ 520 lp/mm at 532 nm wavelength. Then use Equation (2) to get the Bragg angle and wavelength, and put it into Equation (3) in which $c_{s}=1$, Δn=0.017 to get the relationship between the diffraction efficiency of the volume holographic grating and the angle and wavelength. The simulation result is shown in Figure 3.

It can be seen that low diffraction efficiency is obtained at the spatial frequency of 520 lp/mm, and high diffraction efficiencies are obtained at 820 lp/mm, 1120 lp/mm and 1420 lp/mm, which are 82.7%, 94.9% and 97.5%, respectively. The corresponding operation angle ranges are 7.5°, 5.1° and 3.8°, and the corresponding working wavelength bands are 317–757 nm, 427–637 nm and 475–590 nm (all calculated by full width at half maximum). The holographic grating at 820 lp/mm has a wide wavelength band 317–757 nm, which can cover the whole visible band, and has a relatively wide working angle range and high diffraction efficiency. Therefore, we record the VPH with the spatial frequency of 820 lp/mm.

### 2.3. Design and Modeling of the Double-Layer Half-Divided Sunlight Concentration System

**Figure 4:**
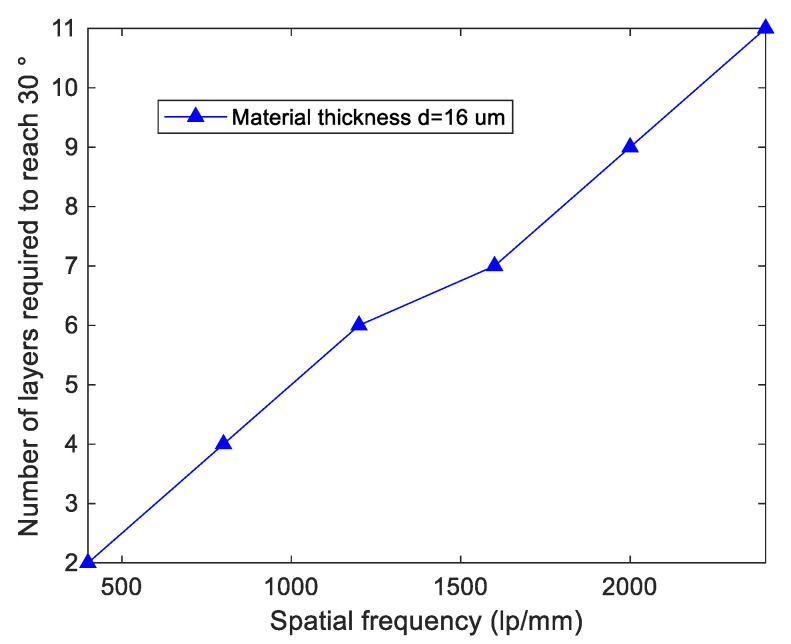
Relationship between the number of layers required to reach 30° and spatial frequency with the material thickness.

To achieve larger operation angle, we require the solar energy concentration system have more layers and more working regions in one layer. Through the Kogelnik coupled wave simulation in Section 2.2, it can be seen that when the material thickness is determined, the working angle of volume holography is related to the spatial frequency of the grating. Assuming that the working angle of the multilayer system is the superposition of the single-layer working system, the relationship between the number of layers required to achieve the working angle range of 30° and the spatial frequency of the multi-layer system is shown in Figure 4.

According to Figure 4, the number of layers required by the system is confirmed to be four under the material thickness for 16 μm and spatial frequency for 820 lp/mm. To avoid interlayer crosstalk in multi-layer concentration system, we design the diffracted light rays by different layers to be concentrated onto different positions to avoid angle multiplexing.

**Figure 5:**
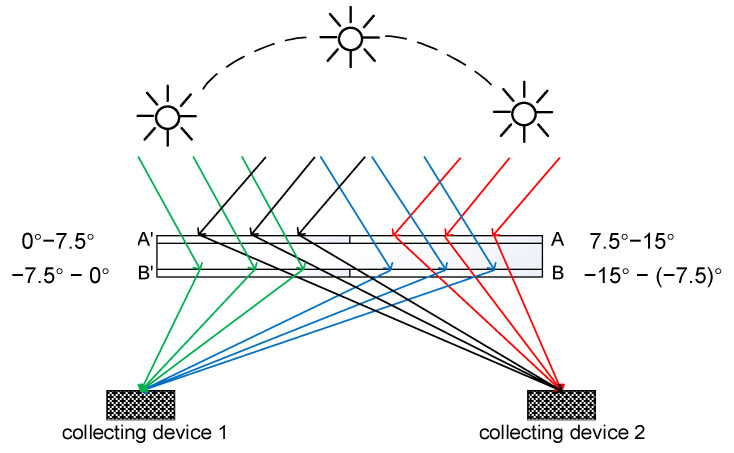
The layout of a crosstalk−reduced double−layer half−divided concentration unit.

After determining the parameters of single-layer volume hologram, we propose a crosstalk-reduced double-layer half-divided concentration unit, whose layout is shown in Figure 5. To display visually the concentration effects, we enlarge the interval between the layers, which is actually small, so that the two layers are closely bonded. Each of the two layers is divided into two holographic gratings, namely A, A′ and B, B′, and there are also two light collecting devices to form a concentration unit. To increase further the concentration ratio and the system concentration area, we can cascade a number of concentration units. The layout is illustrated in Figure 5. For the sunlight incident from 7.5° to 15° (red lines in Figure 5), the grating A concentrates the incident sunlight, and the convergent sunlight transmits B and finally reaches the collecting device 2. For the sunlight incident from 0° to 7.5° (black lines in Figure 5), the sunlight will be converged by A′ and the convergent sunlight will transmit through B′ onto the collecting device 2. For the sunlight incident from −7.5° to 0° (green lines in Figure 5), the sunlight will directly transmit through A′ and be converged by B′ onto the collecting device 1. For the sunlight incident from −15° to −7.5° (blue lines in Figure 5), the sunlight will directly transmit through A and be converged by B onto the collecting device 2. A total of 30° large-angle concentration can be achieved.

As shown in Figure 5, the two layers are glued together to reduce the light energy loss caused by air gap reflection. All of the four holographic gratings are convergent gratings. The gratings diffract sunlight into collecting devices on both sides, respectively, so the incident angle and diffraction angle of each layer designed in this way are not multiplexed, which reduces the interlayer crosstalk. The collecting devices are designed to be on the focal planes of the holographic gratings, so that a large concentration ratio can be achieved.

**Figure 6:**
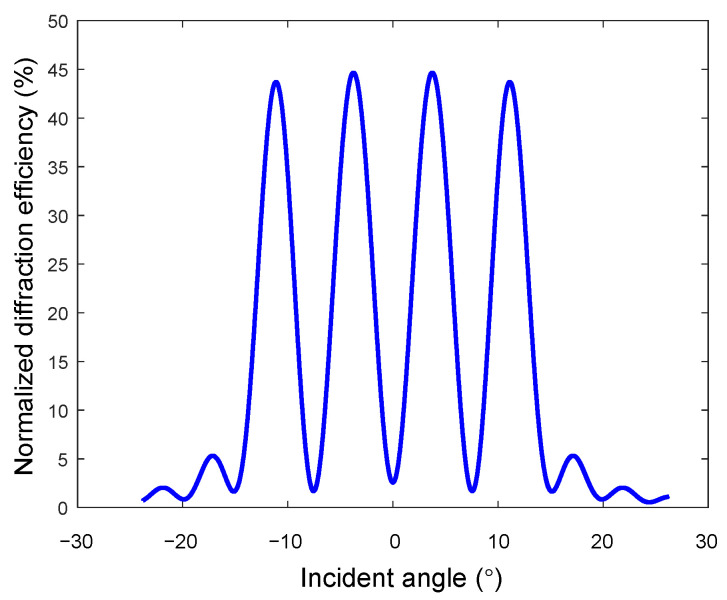
Diffraction efficiency of the double−layer half−divided sunlight concentration system in the whole working angle.

Using the Kogelnik coupled wave theory again, without considering the material’s absorption of light and interlayer interference, the single-layer working angle is 7.5°, the spatial frequency is 820 lp/mm, and the wavelength is 532 nm. Other parameters are the same as in Section 2.2. The simulation result is shown in Figure 6. It can be obtained that the average diffraction efficiency of the condenser system within the working angle of −15° to 15° is 45.63%.

## 3. Recording Holographic Gratings

According to Equation (2), the Bragg angle for a recording wavelength 532 nm can be obtained. It can be seen from Figure 1 that the angles between the object wave or the reference wave with the stripe are both θ, which is also the Bragg angle. The VPH has the largest diffraction efficiency under the Bragg condition. Therefore, the angle between the object wave and the reference wave is twice of the Bragg angle.

**Figure 7:**
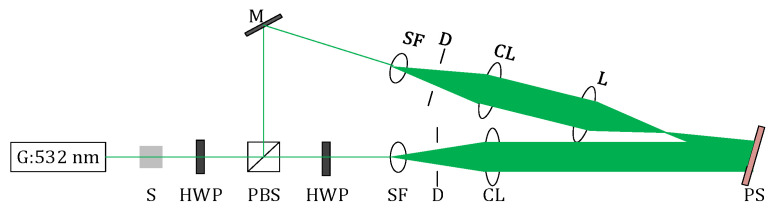
Schematic diagram of the interference recording optical path. S: electronic shutter, HWP: half-wave plate, M: mirror, SF: spatial filter, CL: collimating lens, L: convergent lens, PBS: polarizing beam splitter, PS: photopolymer substrate, D: diaphragm.

The VPH recording light path is shown in Figure 7. The light emitted by a laser having 532 nm wavelength first transmits through an electronic shutter, which is used to precisely control the exposure time. Then, the ratio of transverse magnetic (TM) polarized component to transverse electric (TE) polarized component of the light is controlled by rotating the half wave plate, which is near the laser. Thus, the energy ratio for the object wave to the reference wave can be controlled. The light is then separated into the reference wave with TM mode and the object wave with TE mode by a polarizing beam splitter. The polarization direction of the reference wave is rotated by 90° after transmitting through a half-wave plate, to satisfy the coherent interference conditions with the object wave. Finally, the reference wave and the object wave are spatial filtered and collimated. A convergent lens is used to be the object. A photopolymer substrate is placed at the intersection of the two optical paths for recording VPHs. The four VPHs of the system are recorded by turning the holographic materials, which are located at the rotating platform. The position of the converging lens is kept unchanged, to ensure the same focal length of the four volume holographic gratings.

The exposure intensity for recording the holographic materials is 0.1 mW/cm^2^, and the energy ratio of object and reference waves is 1:1. The dark reaction time is six minutes without preprocessing, and the volume holographic materials generated are most suitable for solar concentration, that is, the operation angle range is 7.5° and the wavelength band is 400–700 nm. After recording, the holographic gratings are UV-cured by using a mercury lamp. The diffraction efficiency of the volume holograms is then measured by an optical power meter.

## 4. Results and Discussion

### 4.1. Optical Properties of a Volume Holographic Grating under Monochromatic Illumination

**Figure 8:**
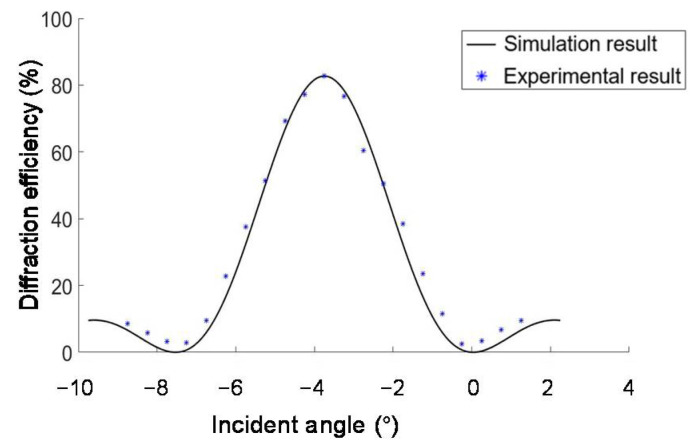
Angle selectivity of the volume−phase hologram (VPH) B′.

The operation angle range of the volume holographic concentration system is related to the angular selectivity of the materials. We measure the diffraction efficiency of a holographic grating B′ under the illumination by collimated 532 nm laser with different incident angles within the operation angle range, and compare the results with the simulation results in Figure 8. It is shown that the experimental results agree with the simulation results. The light energy utilization rate is an important indicator for measuring the concentrating system, which is defined as the ratio of the total amount of light energy at the focus position to the total amount of light energy incident on the concentrating system. We fixed the incident light intensity, rotated the rotating platform, and changed the incident angle to perform 21 sets of experimental measurements. By measuring the diffracted light power and incident light power with a handheld optical power meter (Newport No.840-C), we calculated that the average diffraction efficiency within the angle range was 48.26%.

**Figure 9:**
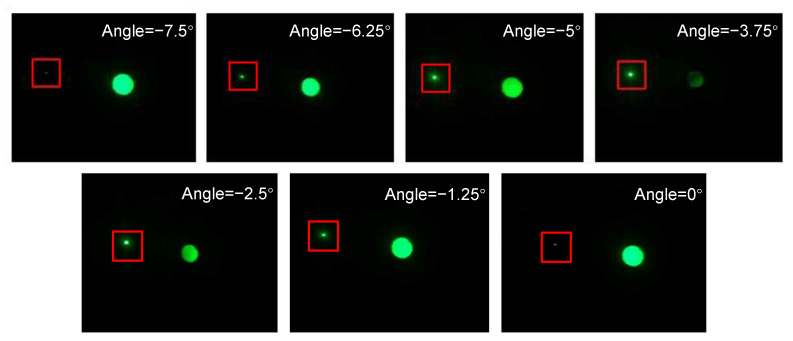
The light distribution on the collecting device 1, when the VPH B′ is illuminated by collimated laser with different incident angles. The small spots in the red rectangles are the focal spots. The big spots are the transmitted light.

We put the sample on the rotating platform (B47-100AN) to change the angle. Within the working angle range, after being irradiated by the 532 nm collimated laser with different incident angles, the light distribution on the focal plane of VPH B′ is shown in Figure 9. It can be seen that, when the illumination angle is −3.75°, which is the Bragg angle of the holographic grating, the energy of the transmitted light is the lowest, which indicates the maximum diffraction efficiency.

The focal spots are collected by a CCD (Charge-coupled Device; MV-EM510M; pixel pitch = 3.45 μm; Microvision; Xian, China), and their sizes are measured. The average diameter of the focal spots is 0.9384 mm, which corresponds to an area of $A_{0}^{\prime }$ = 0.6913 mm^2^. The incident spot average diameter is about 19.2 mm, which corresponds to an area of $A_{0}$ = 289.38 mm^2^. Therefore, the concentration ratio is calculated by $C_{0}=A_{0}/A_{0}^{\prime }=418.8$.

### 4.2. Optical Properties of the Holographic Grating under Sunlight

**Figure 10:**
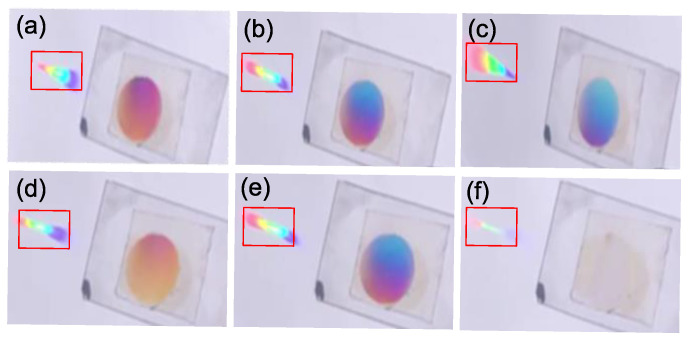
The sunlight concentration results by VPH B′: On focal planes of (**a**) red (**b**) green and (**c**) blue light. With different incident angles: (**d**) deviating a small angle from the Bragg angle, (**e**) Bragg angle, (**f**) deviating a large angle from Bragg angle. The rainbow spots in the red rectangles are the focal spots.

The operation wavelength range of the volume holographic concentration grating B′ is related to the wavelength selectivity of the material. The effect of the VPH B′ under the illumination of sunlight is shown in Figure 10. As a result of the material dispersion, the convergent positions of the light with different wavelengths are different, resulting in a decrease of the concentration ratio under sunlight. The spot under the sun is measured by the scale on the receiving screen. The edge of the spot is distinguished by eyes, twelve measurements are carried and the average value is used as the measurement result. The convergent spot under the Bragg condition illumination of green light at −3.75° can be approximated as a rectangle, the length of which is about 21.5 mm, and the width is about 2.5 mm, so the spot area is about $A_{1}^{\prime }$ = 53.75 mm^2^. The incident spot diameter on the holographic material is about 19.2 mm, and the area is $A_{0}$ = 289.38 mm^2^. Therefore, the concentration ratio under the sunlight is $C_{1}=A_{1}/A_{1}^{\prime }=5.38$. We measured sunlight energy by using a hand-held optical power meter (Newport No.840-C; NEWPORT; 1791 Deere Avenue Irvine; USA). The holographic grating was handheld, and the illumination angle of sunlight was adjusted by rotation of the holographic grating, until the brightest diffraction spot was observed. We measured and calculated the light energy utilization rate in multiple angle ranges to obtain the average light energy utilization rate as 22.16%.

According to Kogelnik coupled wave theory, the Bragg angles for different wavelengths in the sunlight are different, therefore, the focal lengths are different. The focal length for red light is the shortest, and the focal length for blue light is the longest. The convergence results for red, green and blue light are shown in Figure 10a–c. The holographic grating is recorded with 532 nm laser; hence, it has the highest diffraction efficiency at green light, and the convergent spot is the brightest under the condition where the green light is converged. That is also the reason why the transmission light spots on the materials seems to be reddish blue. Illuminated by sunlight with different incident angles, the convergent effects of the holographic grating are shown in Figure 10d–f. Due to the angular selectivity of the holographic grating, the diffraction efficiency decreases sharply with the deviation incident angle from the Bragg angle. The diffraction efficiency substantially approaches zero when the deviation of the Bragg angle is large.

### 4.3. Optical Characteristics of the Double-Layer Half-Divided Concentration System under Monochromatic Illumination

The concentration unit has two layers, each of which is divided into two VPHs, and two light collecting devices. The two layers of the double-layer solar energy concentration system are tightly bonded without gaps. We choose the left half part of the unit (VPH A′ and VPH B′) to analyze the optical properties of the whole system. The left half part of the system is illuminated by collimated 532 nm laser with different incident angles in the operation angle range −7.5° to 7.5°, and then we measure the diffraction efficiency. The diffraction efficiency is obtained by the sum of the diffraction efficiencies at the light collecting devices 1 and 2.

**Figure 11:**
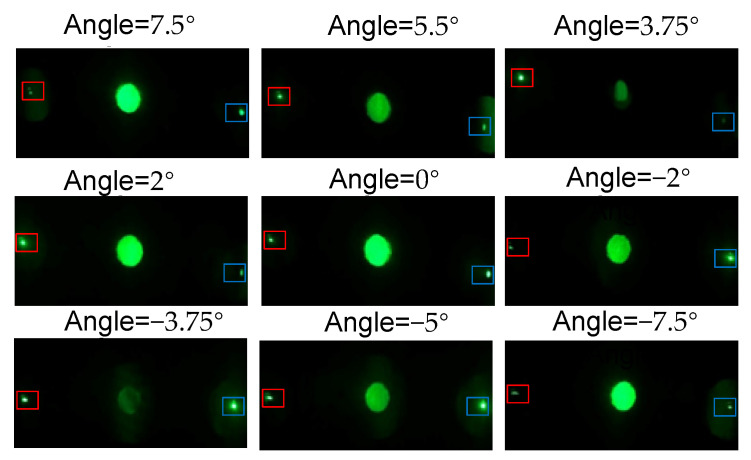
The light distribution on the collecting device plane when the left half of the concentration unit is illuminated by collimated laser with different incident angles. Red and blue rectangles show the position of the collecting device 1 and 2, respectively.

The left half of the unit is illuminated by collimated 532 nm laser with different incident angles in the operation angle range −7.5° to 7.5°, and we capture the light distributions on the plane of the collecting devices, which are shown in Figure 11. It can be seen that, when the illumination angle is −3.75° (the Bragg angle of B′) and 3.75° (the Bragg angle of A′), the energy of the transmitted light is the lowest, which indicates the maximum diffraction efficiency. In addition, the experimental results show that the interlayer crosstalk is small due to the high diffraction efficiency of the gratings. The incident light is, respectively, diffracted by the half unit to the left and right convergent spots corresponding to the light collecting device 1 and 2, as shown in Figure 11.

**Figure 12:**
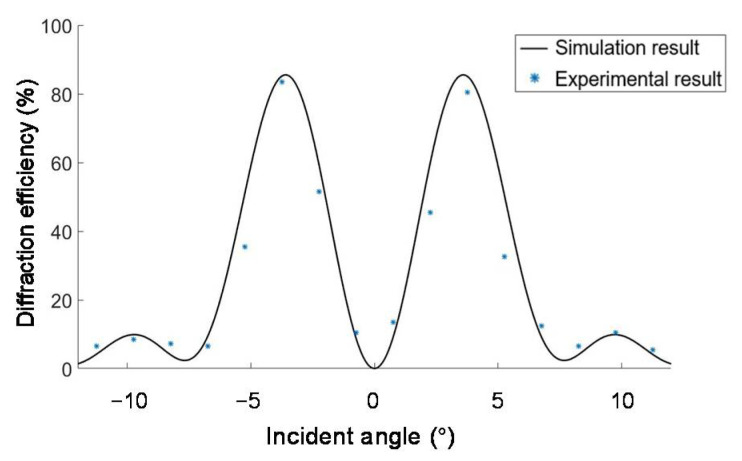
Diffraction efficiency of the half concentration unit composed of A′ and B′ from −7.5° to 7.5° within the operation angle range.

The diffraction efficiency of the left half of the unit is calculated and compared with the simulated results, which are shown in Figure 12. The average value of the difference between the experimentally measured diffraction efficiency and the simulated diffraction efficiency at the same incident angle is 2.9%, which proves the feasibility of the proposed solar energy concentration system. The reduced interlayer crosstalk indicates that the left half of the concentration unit composed of A′ and B′ is well coupled. Due to the small interlayer crosstalk, the diffraction efficiency is almost the same as that of a single VPH. The concentration ratio of the half unit is also measured to be 418.8, which is the same as that of a single VPH.

### 4.4. Optical Properties of the Double-Layer Half-Divided Concentration System under Sunlight Illumination

**Figure 13:**
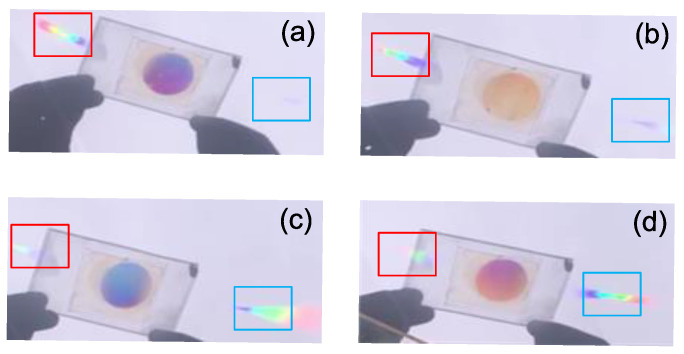
The sunlight concentration results of the left half of the concentration unit (A′ and B′): (**a**) with the Bragg angle of layer A′, (**b**) deviation from the Bragg angle of layer A′, (**c**) deviation from the Bragg angle for layer B′, (**d**) with the Bragg angle of layer B′.

We observe the concentration of sunlight by the half unit (A′ and B′), which is shown in Figure 13. The half concentration unit is rotated by hand to test the concentration effects under the sunlight with different incident angles within the operation angle range. The dispersion of the two VPHs causes the convergent spots to extend and be out of focus along the wavelength band. However, the half unit of the concentration unit also can realize solar energy concentration.

The half concentration unit composed of A′ and B′ has a total operation angle of 15°, which satisfies the Bragg condition of the volume holographic concentration grating A′ when the sunlight is incident at 3.75°, and the maximum diffraction efficiency is obtained at the collecting device 1, as shown in Figure 13a. When the incident angle becomes less than 3.75°, the light intensity at the light collecting device 1 gradually decreases and the light intensity at the device 2 gradually increases. When the incident angle reaches −3.75°, which is the Bragg angle of B′, the light intensity at the light collecting device 2 is the strongest, and the light intensity at the light collecting device 1 is the weakest, as shown in Figure 13d. The double-layer half-divided concentration system can work normally under the illumination of sunlight, which proves the feasibility of the system proposed in this paper applied in large-angle solar energy concentration.

The two layers are both converging VPHs with the same focal length, so the crosstalk between layers is greatly reduced. This can be justified by comparing the single layer’s diffraction efficiency in Figure 8 and the half unit’s diffraction efficiency in Figure 12. Thus, the unit has the same measured concentration ratio as the single hologram, which is 5.38 under sunlight illumination.

### 4.5. Discussion

Compared with the previous work, it can be seen from Table 1 that the concentrating ratio of the volume holographic concentrator designed in this paper is much greater than that of the previous structures, and the concentrating ratio can reach 418.8 under monochromatic light. The light energy utilization and concentration angle also have certain advantages. The structure in this paper is formed by optically irradiating polymers, so long-term exposure to sunlight may cause performance degradation. The preparation process is relatively complicated, and it is difficult to realize large-scale production. Subsequent work can use multicolor laser recording volume holographic structure to increase the utilization rate of light energy and make breakthroughs in the production process to achieve mass production.

## 5. Conclusions

We proposed a multi-layer sunlight concentration system design method, a double-layer, half-divided sunlight concentration system. Only convergent VPHs are used to reduce interlayer crosstalk. Based on the Kogelnik coupled wave theory, the design method provides a trade-off between diffraction efficiency and the number of holographic gratings, and, finally, four VPHs are employed. To make a trade-off between operation angle range and diffraction efficiency, the four VPHs with the same focal length and different Bragg angles are arranged on two layers. With two collection devices placed on the focal points of the VPHs, a double-layer, half-divided sunlight concentration unit is implemented.

The concentration system using VPHs are recorded and tested. The consistent simulation and experiment results for testing the one of the VPHs validate the holographic recording method. The experiment results show that the maximum diffraction efficiency is 87.37% and the operation angle range is 30°. The tested monochromatic concentration ratio of a single VPH achieves 418.8, and the concentration ratio under sunlight is 5.38. The experiment results of light use efficiency are close to the simulation with non-crosstalk, which proves that the interlayer crosstalk is small. This implies that a significant cost reduction in the PV system is possible if the cost differential between the PV cell and holographic material is high [29,30].

## Figures and Tables

**Table 1:** Concentration angle, light energy utilization and concentrating ratio of different structures.

Structure	Concentration Angle (°)	Light Energy Utilization (%)	Concentrating Ratio
by J-H Lee et al. [17]	30	18.22	15
by Castro et al. [18]	120	50	1.5
by Akbari et al. [19]	24	18.64	20
by Bianco et al. [20]	45	13.83	1.8
the proposed volume holographic grating	30	22.16	418.8
